# Supplementary material for: Poly(ADP-Ribose) Polymerase-1 Lacking Enzymatic Activity Is Not Compatible with Mouse Development
Source: Cells. 2023 Aug 16;12(16):2078. doi: 10.3390/cells12162078 (PMC10453916; doi:10.3390/cells12162078)
Supplement: Supplementary file 1 [file cells-12-02078-s001.zip › cells-2494562-supplementary.pdf]

Supplementary figures

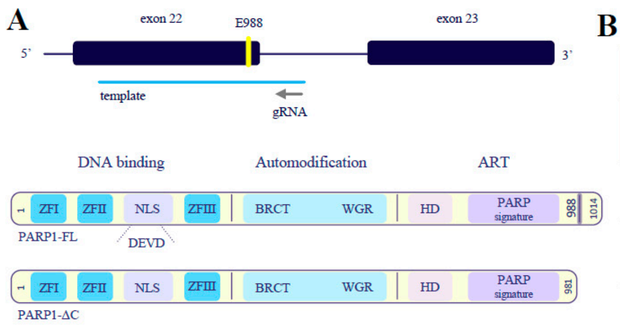

B

Parents		Offspring		
Female	Male	Total	+/+ (%) % expected	+/ΔC (%) % expected
+/+	+/ΔC	142	62 (44%) 50%	80 (56%) 50%
+/ΔC	+/+	31	16 (50%) 50%	15 (50%) 50%

Figure S1. Gene targeting strategy and breeding of mutant animals. (A) Upper panel, the scheme of CRISPR/Cas9-mediated gene targeting of PARP1 aimed to disrupt the C-terminal catalytic domain. The E988 codon is located in exon 22. The template DNA fragment (blue bar) and area for guide RNA (gRNA, arrow) are shown. Lower panel, the protein structure of PARP1 full-length (FL) and PARP1-ΔC are shown. ZF: zinc finger; NLS: nuclear localization signal; BRCT: BRCA1 C-terminal domain, WGR: tryptophan, glycine, arginine-motif domain, HD: helical domain. (B) The genotype frequencies of the offspring from the backcross breeding of PARP1ΔC/+ heterozygotes with PARP1+/+ are summarized. Offspring generated from both male and female PARP1ΔC/+ parents are in expected Mendelian ratios.

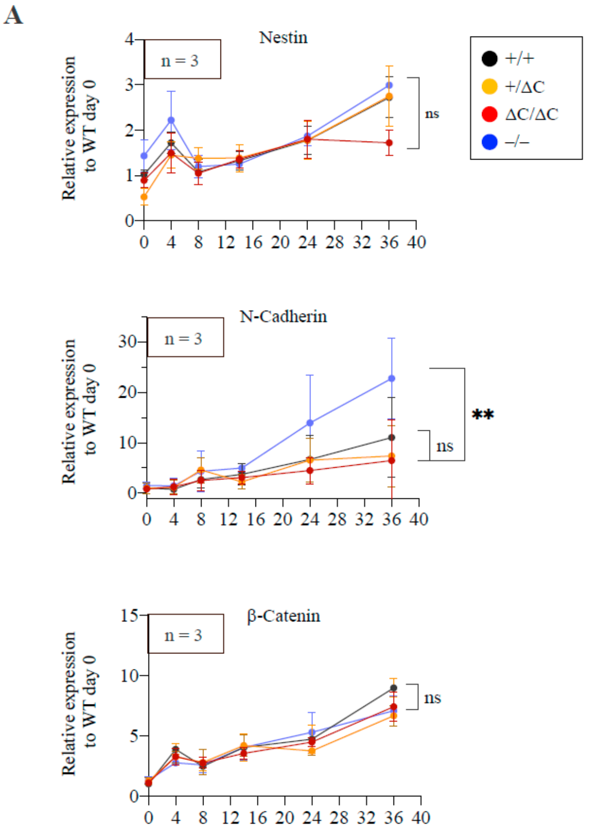

Figure S2. Expression of differentiation markers in PARP1ΔC/ΔC ES cells and EBs. RT-qPCR analysis of (A) Nestin, an early ectoderm marker, (B) N-cadherin, an early mesoderm marker, and (C) β-catenin as an early endoderm marker at 0, 4, 8, 14, 24, and 36 days during EB formation from ES cells of the indicated genotype. n = 3. Statistics by one-way repeated measures nonparametric test,

matched rows with Friedman test, were used to analyse the mean values within individual days, ns, not significant.

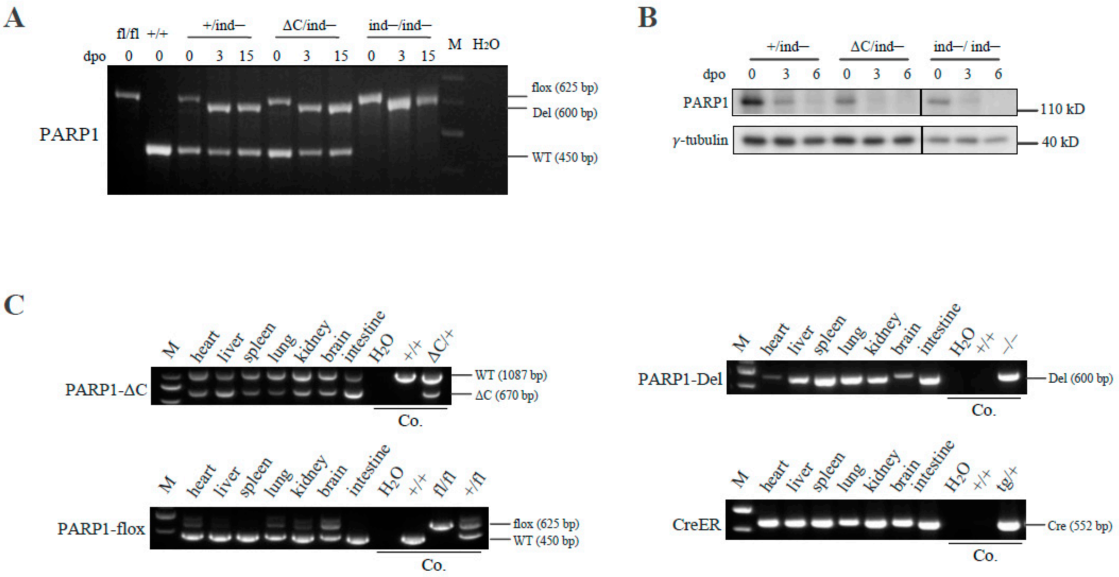

Figure S3. Inducible PARP1ΔC/ind− mice and cells are viable. (A) Genotyping of primary MEF for the PARP1 floxed-allele deletion before (dpo 0) and during (dpo 3 - dpo 6) 4-OHT treatment. The expected (induced) pri-mary MEF genotypes are indicated above the gel. The size of PCR products is indicated next to the gel. M: molecular weight marker. H2O: negative control. (B) Western blot analysis of primary MEFs treated or untreated with 4-OHT. The genotypes expected after the 4-OHT treatment are indicated above the gel. The polyclonal ab against PARP1 N-terminal part (DEVD) detected PARP1 full-length protein, which disappeared in PARP1ind−/ind− and PARP1ΔC/ind− at 6dpo. Note, as ex-pected, the expression of PARP1-ΔC protein in PARP1ΔC/ind− MEFs is barely detectable. γ-tubulin serves as a loading control. (C) The genotyping results for the indicated organs of PARP1ΔC/fl CreERtg/+ mice, which were treated with intraperitoneal injections of tamoxifen (75 mg/kg/day) for 4 consecutive days. The genotyping of the allele is indicated to the left, and the size of the allele by PCR is indicated to the right of the gel. WT: PARP1 wild-type allele, ΔC: PARP1-ΔC allele, flox: PARP1 floxed allele, Del: PARP1 deletion allele, Cre: CreER transgene.

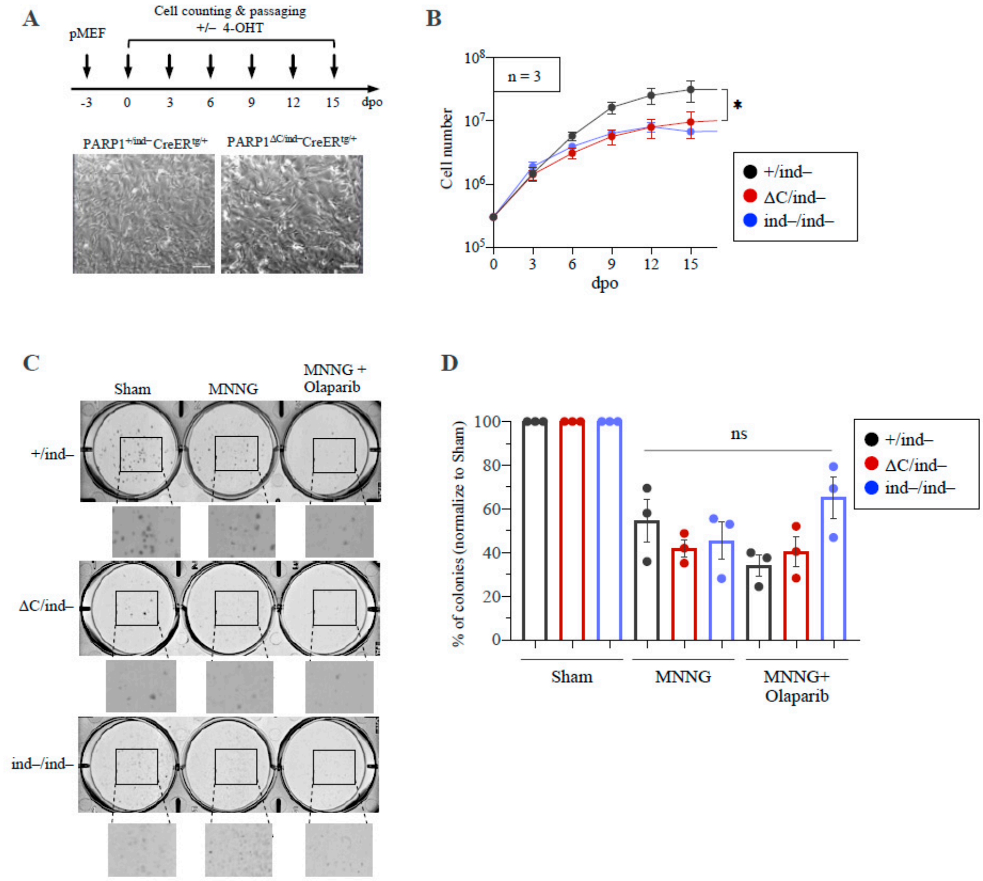

Figure S4. Inducible PARP1ΔC/ind− mice and cells are viable but sensitive to genotoxic treatment. (A). Upper panel, the protocol for primary MEF treatment with 4-OHT and, lower panel, representative images of primary MEFs (passage 7) at 12 days post-4-OHT (dpo). The arrows in the protocol indicate cell counting and passaging (three hundred thousand cells/well) of the time point of dpo. Lower panel, the brightfield images of MEFs of the indicated genotype with 4-OHT treatment. The scale bar: 100 µm. (B) The cumulative growth curves of primary MEFs of the indicated genotypes generated after 4-OHT treatment. 4-OHT was present in the culture medium during the whole experiment. Statistics by one-way repeated measures nonparametric test, matched rows with Friedman test were used to analyse the mean values within individual days, ns, not significant. (C) Colony formation assay. 400 primary MEF cells per well were seeded in a 6-well plate and treated 24 hr later. Then the cells were re-passaged in a new 6-well plate after another 24 hr and grew for 5 days before fixation and counting. (D) Quantification of the number of colonies in (C). ns, not significant.
